# Integration of whole genome sequencing into a healthcare setting: high diagnostic rates across multiple clinical entities in 3219 rare disease patients

**DOI:** 10.1186/s13073-021-00855-5

**Published:** 2021-03-17

**Authors:** Henrik Stranneheim, Kristina Lagerstedt-Robinson, Måns Magnusson, Malin Kvarnung, Daniel Nilsson, Nicole Lesko, Martin Engvall, Britt-Marie Anderlid, Henrik Arnell, Carolina Backman Johansson, Michela Barbaro, Erik Björck, Helene Bruhn, Jesper Eisfeldt, Christoph Freyer, Giedre Grigelioniene, Peter Gustavsson, Anna Hammarsjö, Maritta Hellström-Pigg, Erik Iwarsson, Anders Jemt, Mikael Laaksonen, Sara Lind Enoksson, Helena Malmgren, Karin Naess, Magnus Nordenskjöld, Mikael Oscarson, Maria Pettersson, Chiara Rasi, Adam Rosenbaum, Ellika Sahlin, Eliane Sardh, Tommy Stödberg, Bianca Tesi, Emma Tham, Håkan Thonberg, Virpi Töhönen, Ulrika von Döbeln, Daphne Vassiliou, Sofie Vonlanthen, Ann-Charlotte Wikström, Josephine Wincent, Ola Winqvist, Anna Wredenberg, Sofia Ygberg, Rolf H. Zetterström, Per Marits, Maria Johansson Soller, Ann Nordgren, Valtteri Wirta, Anna Lindstrand, Anna Wedell

**Affiliations:** 1 Department of Molecular Medicine and Surgery, Karolinska Institutet, Stockholm, Sweden; 2 Centre for Inherited Metabolic Diseases, Karolinska University Hospital, Stockholm, Sweden; 3 Science for Life Laboratory, Department of Microbiology, Tumour and Cell Biology, Karolinska Institutet, Stockholm, Sweden; 4 Department of Clinical Genetics, Karolinska University Hospital, Stockholm, Sweden; 5 Department of Women’s and Children’s Health, Karolinska Institutet, Stockholm, Sweden; 6 Department of Medical Biochemistry and Biophysics, Karolinska Institutet, Stockholm, Sweden; 7 Science for Life Laboratory, School of Engineering Sciences in Chemistry, Biotechnology and Health, KTH Royal Institutet of Technology, Stockholm, Sweden; 8 Department of Clinical Immunology and Transfusion Medicine, Karolinska University Hospital, Stockholm, Sweden; 9 Science for Life Laboratory, Department of Molecular Medicine and Surgery, Karolinska Institutet, Stockholm, Sweden

**Keywords:** Whole genome sequencing, Monogenic disease, Single nucleotide variant, Clinical diagnostics

## Abstract

**Background:**

We report the findings from 4437 individuals (3219 patients and 1218 relatives) who have been analyzed by whole genome sequencing (WGS) at the Genomic Medicine Center Karolinska-Rare Diseases (GMCK-RD) since mid-2015. GMCK-RD represents a long-term collaborative initiative between Karolinska University Hospital and Science for Life Laboratory to establish advanced, genomics-based diagnostics in the Stockholm healthcare setting.

**Methods:**

Our analysis covers detection and interpretation of SNVs, INDELs, uniparental disomy, CNVs, balanced structural variants, and short tandem repeat expansions. Visualization of results for clinical interpretation is carried out in Scout—a custom-developed decision support system. Results from both singleton (84%) and trio/family (16%) analyses are reported. Variant interpretation is done by 15 expert teams at the hospital involving staff from three clinics. For patients with complex phenotypes, data is shared between the teams.

**Results:**

Overall, 40% of the patients received a molecular diagnosis ranging from 19 to 54% for specific disease groups. There was heterogeneity regarding causative genes (*n* = 754) with some of the most common ones being *COL2A1* (*n* = 12; skeletal dysplasia), *SCN1A* (*n* = 8; epilepsy), and *TNFRSF13B* (*n* = 4; inborn errors of immunity). Some causative variants were recurrent, including previously known founder mutations, some novel mutations, and recurrent de novo mutations. Overall, GMCK-RD has resulted in a large number of patients receiving specific molecular diagnoses. Furthermore, negative cases have been included in research studies that have resulted in the discovery of 17 published, novel disease-causing genes. To facilitate the discovery of new disease genes, GMCK-RD has joined international data sharing initiatives, including ClinVar, UDNI, Beacon, and MatchMaker Exchange.

**Conclusions:**

Clinical WGS at GMCK-RD has provided molecular diagnoses to over 1200 individuals with a broad range of rare diseases. Consolidation and spread of this clinical-academic partnership will enable large-scale national collaboration.

**Supplementary Information:**

The online version contains supplementary material available at 10.1186/s13073-021-00855-5.

## Background

Diagnostics of genetic diseases are currently being revolutionized, due to breakthroughs in sequencing technology and data analysis. The potential to transform clinical medicine using genomics is high, especially within the realm of rare diseases. Rare diseases constitute a large and heterogeneous group of diagnoses that includes more than 8000 distinct conditions [1, 2] of which the vast majority have a genetic basis. Each individual disease is rare, but when considered as a group, rare diseases are common with a total prevalence of approximately 6–8% [3, 4].

The prevalence of rare diseases is highly variable. A few of these diseases are relatively common with a prevalence above 1/20,000, while the vast majority are very rare [5].

The clinical presentation of these diseases includes a broad diversity of symptoms and signs, ranging from mild features affecting only part of the body to severe manifestations involving multiple organ systems. The nervous system is commonly affected, resulting in symptoms such as intellectual disability (ID), neuropsychiatric diseases, epilepsy (EP), and motor dysfunction. Age of onset ranges from the prenatal period into late adulthood, and it is estimated that half of the affected cases are referred by a pediatrician. Many of the rare diseases cause chronic disabilities with significant impact on the lives of affected individuals and their families as well as on the healthcare system [6]. In order to optimize treatment and care as well as genetic counseling regarding prognosis and recurrence risks, establishing the specific diagnosis is crucial. For many diseases, such as inborn errors of metabolism (IEM), treatments are available in the form of specific diets, recombinant enzymes, small molecule drugs, or antisense technology. Initiation of treatment in early disease stages can sometimes prevent serious handicaps or early death, making rapid diagnostics essential.

Implementation of genome sequencing into the clinic is dependent on each country’s specific organization of healthcare and academia. Swedish public healthcare is decentralized to 21 regions and is financed primarily through taxes levied at the same level. Public funding for research and innovation, on the other hand, is a governmental responsibility. This creates structural limitations for work across organizations and hinders systematic integration of innovations into healthcare. Swedish legislation does not allow sharing of patient data between public healthcare regions, complicating national coordination. Healthcare is also strictly subdivided into functional units that most often follow clinical disciplines, each with a detailed control of management, which adds to the difficulties of establishing creative, multidisciplinary environments with the possibility to adopt the latest technologies.

Genome sequencing requires infrastructure and expertise on a level beyond the scope of public healthcare funding and is thus critically dependent on academia. Science for Life Laboratory (SciLifeLab) is a national infrastructure funded by the Swedish government with the mission to provide high-throughput bioscience through technical platforms, including massively parallel sequencing (MPS). SciLifeLab started out in 2010 as a joint effort between four universities: Karolinska Institutet, KTH Royal Institute of Technology, Stockholm University, and Uppsala University. Today, SciLifeLab supports research activities at all major Swedish universities. Many international genome centers have been established and several large-scale international and national sequencing projects have been launched [7–13] but clinical integration is lagging behind. In order to enable integration of genomics into rare disease healthcare, we established Genomic Medicine Center Karolinska-Rare Diseases (GMCK-RD), an academic-clinical collaboration between the SciLifeLab Clinical Genomics facility and public healthcare in the Stockholm region to implement whole genome sequencing (WGS) in the diagnostics of rare diseases. No precedence exists for using academic infrastructure in public healthcare as these two governance systems are completely different. Despite the challenges described, an integrated, translational environment has been established where bioinformatics tools, workflows, and decision support systems are continuously developed and improved by multidisciplinary teams including broad technical, experimental, and clinical expertise. This has enabled customized analyses, sharing, and interpretation of genomics data all the way to rapid clinical translation through three different clinics at the Karolinska University Hospital (Clinical Genetics, Center for Inherited Metabolic Diseases, and Clinical Immunology) focused on different disease groups. We report the results from the first 5 years using clinical WGS, which has been gradually implemented in a bottom-up approach, by stepwise addition of new components to the workflow. More than 4400 clinical samples have been analyzed, resulting in a large number of cases receiving rare and specific molecular diagnoses. In order to consolidate and spread this concept to additional disease groups and healthcare regions, and to enable large-scale, national, prospective studies with more in-depth analyses of population-level clinical genome data, the decentralized organization of Swedish healthcare needs to be challenged. Part of this work was previously presented as a conference abstract [14].

## Methods

### Detailed descriptions of partners in GMCK-RD

GMCK-RD is organized as a trans-clinic unit at the Karolinska University Laboratory (Karolinska University Hospital, Stockholm, Sweden), interconnecting SciLifeLab Clinical Genomics facility and three different clinics (Clinical Genetics, Centre for Inherited Metabolic Diseases, and Clinical Immunology). These three clinics are responsible for the vast majority of all clinical genetic testing in the Stockholm healthcare region, and GMCK-RD performs all clinical WGS for patients in this region and nationally for some disease groups; currently, ~ 2000 samples are sequenced annually. Each partner contributes with unique in-depth knowledge in their specialty area. In brief, *Clinical Genetics* provides diagnostic service and genetic counseling to patients from the Stockholm region with, or at risk of, a broad range of genetic disorders. The center offers diagnostic testing for symptomatic individuals as well as carrier testing/pre-symptomatic testing for individuals at risk. For families with an increased risk of having a child with a genetic disorder, the center offers targeted prenatal diagnostics and/or pre-implantation genetic diagnostics. Furthermore, the center performs genetic trisomy-screening of ongoing pregnancies, by non-invasive prenatal testing and/or invasive testing on samples from chorionic villus biopsy or amniotic fluid. *The Centre for Inherited Metabolic Diseases* is an integrated expert center where clinical specialists work closely together with experts in laboratory medicine, combining clinical genetics, clinical chemistry, pediatrics, neurology, and endocrinology. The center serves the whole Swedish population with diagnostics and expert advice on IEM and has a broad arsenal of biochemical investigations designed to detect defects in intermediary metabolism. For investigation of mitochondrial diseases, mitochondria are isolated from muscle biopsies for analysis of ATP production using a range of substrate combinations, determination of activities of respiratory chain complexes, and analysis of nuclear and mitochondrial DNA. The center also performs the national neonatal screening program, currently comprising 25 treatable diseases. Dried blood spot samples (DBS) are stored in the phenylketonuria (PKU) biobank, currently holding around 4.6 million of Sweden’s 10.2 million inhabitants. *Clinical Immunology* performs primary immunodeficiency (PID) genetic diagnostics nationally. The center also performs cellular analyses for immunodeficiencies, as well as being the transplantation center for Stockholm, performing workup and follow-up after hematopoietic stem cell and solid organ transplantations. Finally, the *SciLifeLab Clinical Genomics facility* provides an infrastructure and expertise for clinical massively parallel sequencing, covering data generation, bioinformatic analysis, and software development, including decision support systems.

Our joint efforts have been aimed at introducing WGS as a comprehensive, first-line diagnostic test including rapid WGS (rWGS) in acutely presenting and intensive care individuals. Our clinical genomics workflow includes phenotype-specific gene panels as well as an online mendelian inheritance in man (OMIM) morbid gene panel for patients with complex phenotypes. Cases are analyzed as either singletons or trios (i.e., patient and parents). The integrated collaborative environment of GMCK-RD enables us to match genotype data with phenotypic information such as detailed clinical assessment, imaging data, biochemical measurements, and immunophenotyping.

### General process for clinical whole genome sequencing at GMCK-RD

The infrastructure and close proximity of key resources at the Karolinska University Hospital-Karolinska Institutet-SciLifeLab has for us been vital to a successful integration of genomics into healthcare.

The necessary components that have enabled clinical integration are detailed below.

#### Patient recruitment

**Fig. 1:**
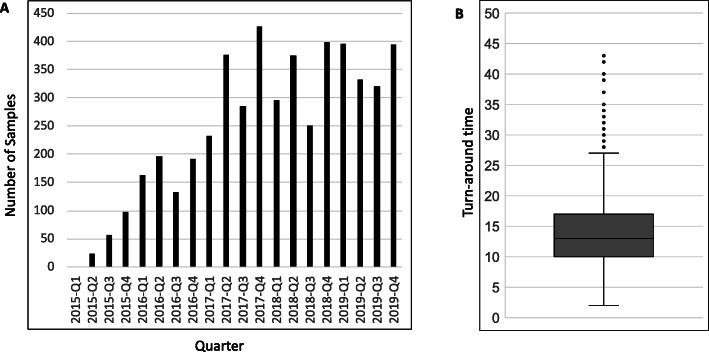
**a** Number of individuals whose genomic DNA were sequenced by WGS at GMCK-RD per quarter between years 2015 and 2019. **b** Turnaround time for sequencing ranged from 2 to 43 days with a median of 13 days

All patients were initially referred for clinical diagnostic testing between the years 2015 and 2019. During this period, 3219 rare disease cases have been analyzed by clinical WGS through GMCK-RD, including 608 trio/family analyses amounting to a total of 4437 individuals sequenced (Table 1; Fig. 1).

**Table 1:** In total, 3750 panels were analyzed by the 15 different teams in GMCK-RD. In total, there were 34% (*n* = 1285) positive findings. Abbreviation in parenthesis refers to the sheet in Additional file 2: Table S6, where the contents of the gene panels are described in detail

Panels	Number of analyses	Solved (%)	Number of genes (from 2015 to 2019)
Metabolic including mitochondrial diseases (singleton analysis) (IEM)	849	274 (32%)	610–870
Neuromuscular and ataxia disease (singleton analysis) (NMD)	455	189 (42%)	499–622
Targeted gene panel (HPO etc.) (singleton analysis)	429	124 (29%)	Variable
Severe infantile epilepsy (trio analysis) (EP)	327	101 (31%)	138–353
Immunology (singleton analysis) including neutropenia (PID)	300	88 (29%)	26–425
OMIM morbid gene panel (trio analysis) (OMIM-morbid)	281	116 (41%)	3103–3921
Intellectual disability and malformation syndromes (singleton analysis) (ID)	304	119 (39%)	885–987
Connective tissue disease (singleton analysis) (CTD)	245	68 (28%)	101–118
Skeletal dysplasia (singleton analysis) (SKD)	212	115 (54%)	376–468
Inherited cancer (singleton analysis) (IC)	147	29 (20%)	116–154
Disorder of sex development (singleton analysis) (DSD)	68	17 (25%)	118–130
Pediatric hepatology (singleton analysis) (PEDHEP)	53	18 (34%)	58–124
Ciliopathy (singleton analysis) (CIL)	36	19 (53%)	168–195
Neurodegenerative disorders (singleton analysis) (NDD)	32	6 (19%)	81–88
Fetal hydrops (singleton analysis) (FETHYD)	12	6 (50%)	57–104

All analyses were ordered as clinical tests and all patients were clinically assessed by the referring physician. For some disease groups, referring physicians are active members of the specialized teams, facilitating identification of patients, interpretation of genomic findings in relation to the clinical picture, and rapid translation all the way to individualized patient management. The assessment entails a thorough phenotyping regarding symptoms and signs, as well as clinical investigations, which often include biochemical testing, imaging studies, neurophysiological tests, evaluation of cognitive level and potential neuropsychiatric diseases, histopathologic tissue studies, and more. In addition, a pedigree for each patient has been established. This information is recorded in the patient’s medical records and can manually be accessed from those.

Prior to WGS, patients and/or their legal guardians have received pretest information and given their consent to clinical testing.

#### Data generation

In most cases, DNA was extracted from blood samples (*n* = 4214; 95%), but in some cases, other tissues were used such as muscle biopsy (*n* = 152), DBS from the newborn screening biobank (*n* = 5), or fetal tissue (*n* = 66). For suspected mitochondrial diseases, the preferred tissue of analysis is muscle biopsy specimens as this allows detailed biochemical evaluation of respiratory chain function together with analysis of both nuclear and mitochondrial DNA. Mutations in mitochondrial DNA (mtDNA) show variable degrees of heteroplasmy in different tissues, with muscle tissue representing the gold standard for diagnostics, and hence analysis of mtDNA was restricted to these cases.

In all cases, extracted DNA was converted to sequencing libraries using a PCR-free paired-end protocol (either Illumina TruSeq DNA PCR-free for > 1000 ng input or Lucigen NxSeq AmpFREE Low DNA > 100 ng). Sequencing was first done using the Hiseq X Ten (*n* = 2866) and from December 2018 on the Illumina NovaSeq 6000 (*n* = 1571) platforms aiming at 30x median coverage. Based on performance assessments done using Genome-in-a-bottle reference material, every sample was sequenced until at least 26x coverage (typically 275–325 M read pairs) was obtained.

To ensure there are no sample mix ups during the WGS processing, an aliquot of the extracted DNA was genotyped for 51 SNPs using MassARRAY technology (Agena Biosciences), and the obtained SNP fingerprint compared to genotypes called from the WGS data. The SNPs have been chosen to have high minor allele frequency in the Swedish population and cover all autosomes (Additional file 1: Table S1).

#### Bioinformatics analysis

**Fig. 2:**
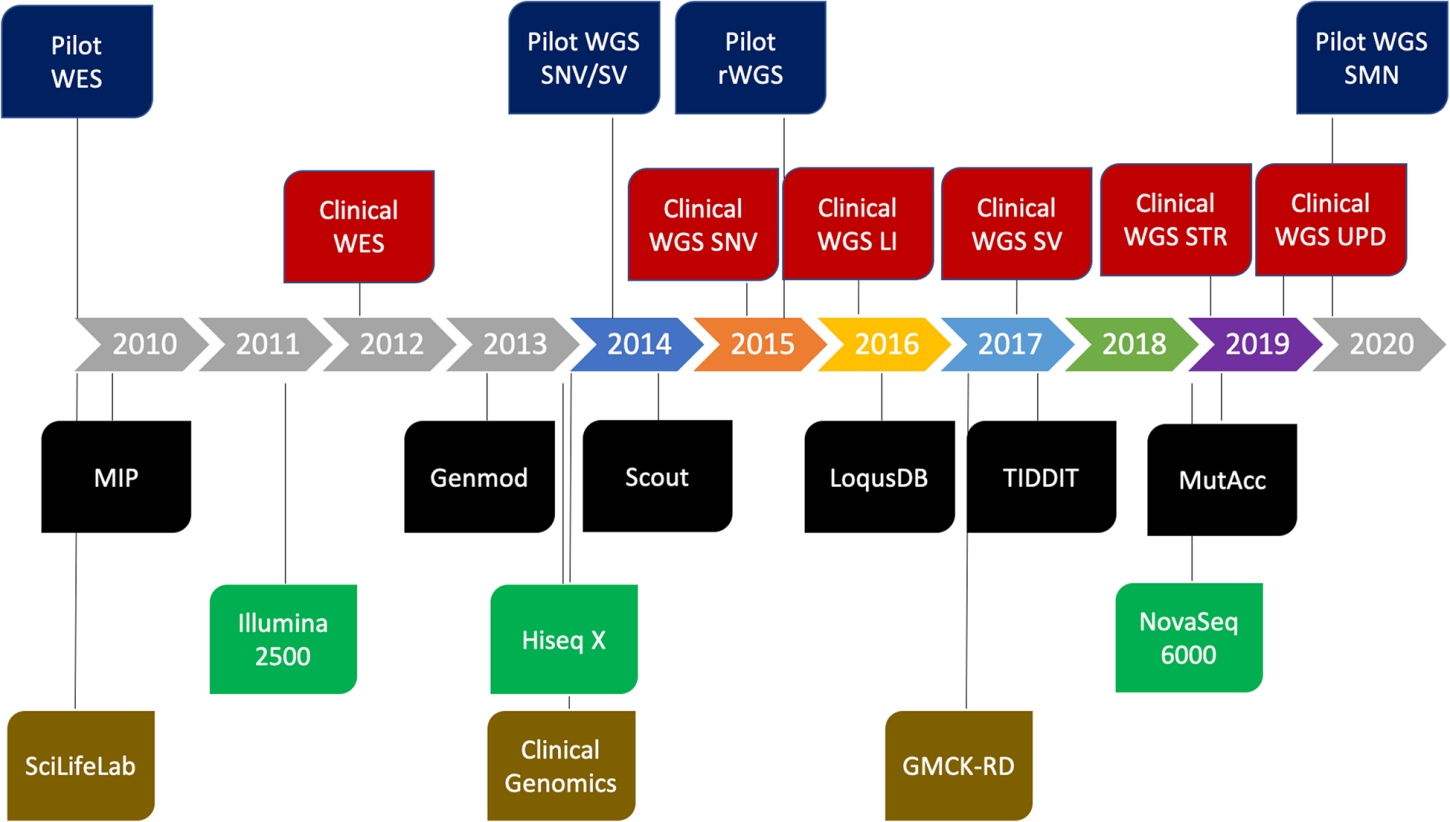
Massively parallel sequencing (MPS)-based diagnostics at the Genomic Medicine Center Karolinska. Timeline showing the integration of genome sequencing into healthcare by gradually adding novel components to the workflow. Blue = MPS pilots; red = clinical routine analysis; black = bioinformatic softwares developed in-house; green = sequencing instruments; gold = organizational structures/resources. GMCK-RD, Genomic Medicine Center Karolinska-Rare Diseases; LI, low input DNA; MIP, mutation identification pipeline; rWGS, rapid whole genome sequencing; Scilifelab, Science for Life Laboratory; SNV, single nucleotide variant; SMN, copy number identification of SMN1 and SMN2 genes; STR, short tandem repeat; SV, structural variant; UPD, uniparental disomy; WES, whole exome sequencing; WGS, whole genome sequencing

**Fig. 3:**
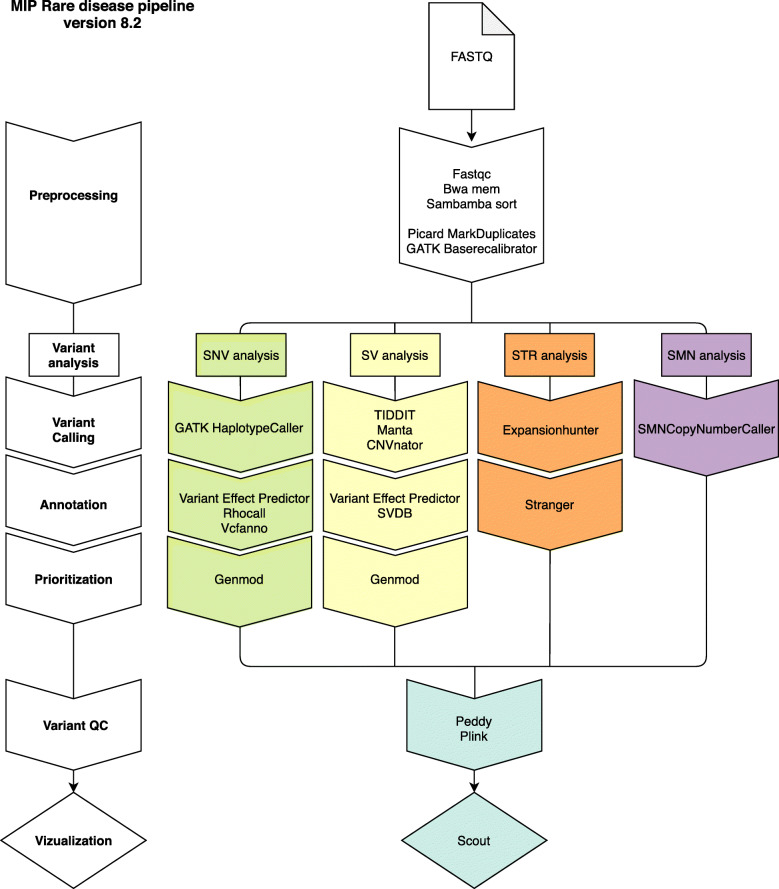
Schematic illustration of the different components in our current bioinformatic pipeline (MIP 8.2). First (in white), FASTQ data is aligned to the human reference genome. Next, different variant types are called including SNV/INDEL (green), SV (yellow), STR (orange), and SMN (purple). Each variant type is then annotated and prioritized before it is vizualized in Scout

The resulting WGS data was processed using a combination of pre-existing and custom-developed open-source tools (Additional file 1: Supplementary Methods) using the Mutation Identification Pipeline framework (MIP) [15]. The analysis was initially optimized for the detection of single nucleotide variants (SNVs) and insertions and deletions (INDELs). Gradually, analyses of structural variants, uniparental disomy (UPD), repeat expansions, and copy number identification of the *SMN1* and *SMN2* genes have been developed and added to the analysis workflow (Figs. 2 and 3). For more detailed information on bioinformatic softwares and steps in MIP, see Additional file 1: Figure S1.

The current version, MIP 8.2 (https://github.com/Clinical-Genomics/MIP), employs mapping to hg19 (ds5) with bwa [16], a GATK [17] best practice variant calling workflow and annotation and prioritization of called variants. For structural variant (SV) calling, Manta [18], CNVnator [19], and TIDDIT [20], variant calls are combined using SVDB [21]. Variant annotation from static databases as well as merging calls from multiple SV callers is performed by SVDB [21]. Repeat expansions at known loci are called with ExpansionHunter [22] and annotated using Stranger [23] (Additional file 1: Supplementary Methods). Mitochondrial variants were called using GATK. During the period 2015–2019, other callers have been used as well, but their inclusion has been discontinued as a consequence of continuous evaluation of performance (sensitivity, specificity, computational cost, etc.). See Fig. 2 for an overview of how the different callers were introduced over time and Additional file 1: Supplementary Methods for details.

After variant calling, variants (including SNVs, INDELs, and SVs) from one individual per family were loaded into a local database—LoqusDB [24], generating an in-house variant database that allows for efficient detection of the rare patient cohort-specific variants complementing global frequency databases, e.g., gnomAD [25]. Furthermore, it enables annotation and down prioritization of local systematic artifacts originating from the sequencing and bioinformatic analysis. For trio analyses, the expected familial relationships are confirmed by using Peddy [26] and Plink [27].

Regions with insufficient sequence data coverage in genes and transcripts for each in silico gene panel were analyzed using the tool Chanjo [28]. Chanjo produces both a clinical report of the mean coverage at different coverage depths and the number of completely covered transcripts at a specified coverage threshold. The Chanjo database can also be used for more in-depth coverage analysis.

Called variants were then annotated using VEP [29], Vcfanno [30], and Genmod [31] (Additional file 1: Supplementary Methods) to acquire an information-rich dataset enabling further automated bioinformatic variant prioritization in respect to rare disease diagnostics.

All called and annotated SNVs, INDELs, and SVs were given a prioritization score by applying a rank model based on weighted sums, by the tool Genmod [31] (Additional file 1: Supplementary Methods). Currently, SNVs and INDELs are scored using one combined rank model, while SVs are scored by a different rank model. Multiple parameters are taken into account, e.g., Mendelian inheritance pattern, conservation, rarity, and predicted protein impact. Currently, the rank model does not use phenotype data from the subjects included in the analysis. Detailed information about the rank models is available on GitHub [32]. The prioritization score is applied in the final step of the bioinformatic analysis to present to the investigator the most likely disease-causing variants according to the rank model applied. The rank score effectively reduces the number of potential disease-causing variants from hundreds of thousands, or even millions in whole genome analyses, down to a manageable prioritized small set of candidates for further manual investigation in the clinical variant interpretation step. However, all prioritized variants are kept and can be made available in the interpretation process if required.

##### Clinical variant interpretation

The cross-clinic work within GMCK-RD is organized into specialized teams where variant interpretation is performed by clinical laboratory geneticists together with physicians from the three clinics in GMCK-RD who are experts in their specific area. Altogether, the expert teams are responsible for interpreting 15 phenotype-specific gene panels (Table 1; Additional file 2: Table S6).

Each team is responsible for compiling panels of genes (in silico phenotype-specific gene panels) relevant for their clinical specialty and these are updated regularly, typically 2–4 times annually [33]. Genes are gathered from knowledge, commercial gene tests, literature, and own research with the requirement that the gene has been clearly linked to disease by publication in a peer-reviewed journal. Diagnostic-grade gene panels for corresponding diseases available through the Genomics England PanelApp were generally included [34]. The gene panels were imported into a graphical user interface for massively parallel sequencing (MPS) data and metadata, Scout [35], and used for selecting and scoring variants within the specific panel. Customized panels were also created by the Scout software using patient-specific human phenotype ontology (HPO) terms [36] that were entered into the system manually. This was used both as primary analysis for cases that did not fit one of the disease-specific gene panels and as a secondary analysis in some cases where the disease-specific gene panel could not detect a causative variant.

The prioritized variants in the requested gene panel are uploaded into Scout [35], a clinical decision support system that provides a unified and intuitive interface for rapid integration in a diagnostic setting. Scout is accessed via a standard web browser and organizes cases for clinical interpretation, enabling collaboration within and between teams. Each variant call in Scout is richly annotated using both common and custom annotations and can be inspected, filtered, and classified. Scout enables sharing of data between teams within GMCK-RD as well as with the global community through ClinVar, Beacon, and Matchmaker Exchange, where the GMCK node is denoted “patient Matcher.”

The clinical filtering and interpretation are done in three steps.

- Firstly, analysis is performed focusing on medically relevant variants given the suspected disease of the patient. To this end, the genome data is filtered in silico for pre-compiled clinically relevant gene panel(s) depending on the clinical presentation of the patients. Analysis is mostly done as singleton (patient only) but in patients with a complex phenotype that are highly heterogeneous, such as congenital syndromes, trios are preferred. In such cases, it is possible to analyze very large gene panels such as the entire OMIM morbid gene panel including 3959 genes. Trios are also preferred for disease groups with a high proportion of de novo variants, such as infantile epilepsy.
- Secondly, when appropriate, the data can be shared and reanalyzed by another team within GMCK-RD. This is particularly important for patients with more complex clinical presentations matching several medical areas.
- Finally, if a molecular finding is still not obtained and the suspicion of a rare genetic disease is high, the patients/families are offered a research-setting analysis where the whole genome is considered.

In steps 2 and 3, variants may be shared internationally through ClinVar, Beacon, and Matchmaker Exchange. For this purpose, the Scout interface has built in modules, enabling different levels of data sharing from gene to variant and with or without phenotype information (Additional file 1: Supplementary Methods).

##### Confirmation of detected genomic aberrations by a secondary method

Until 2019, all reported variants from WGS analysis were verified using a secondary method: in the case of SNVs and INDELs by Sanger sequencing and in the case of deletions/duplications (> 50 bp) using MLPA, clinical microarray, or breakpoint junction PCR. Since the proportion of false positives (wrong genotype call) for SNVs was perceived to be low based upon experience, WGS data from 721 consecutive findings both true- and false-positive SNVs and INDELs was further analyzed. In short, these highlighted aberrations were collected from Scout and analyzed using a set of easily obtainable parameters (sequence depth, genotype quality score, GATK filter status, presence in segmental duplications, and manual inspection in integrative genomics viewer (IGV) [37] or IGV.js [38]). The results from the analysis were used to set criteria that had to be fulfilled in order to report the aberration without verification using a secondary method and excluding the risk of reporting false-positive results.

##### Reporting of results

Interdisciplinary rounds were conducted within each team. Results were reported out to the referring physician and patients/families were offered genetic counseling when positive findings were made. As clinical experts from relevant disciplines were involved in each team, sometimes including the referring physician, translation of genetic findings into individualized treatment was enabled. In negative cases, where the suspicion of a rare genetic condition remained high, a renewed referral was recommended within 6–12 months for high suspicion of more acute conditions, and otherwise 3–5 years for reanalysis of genome data.

Regarding summarization of the WGS results from the three clinics, data were combined from locally stored spreadsheets with compilation of results together with extraction of data from a laboratory information management system (STARLIMS, Abbott Laboratories, IL, USA).

##### Continuous quality assurance, development, and innovation

To ensure high-quality analyses, a set of quality assurance steps have been implemented throughout the clinical diagnostic workflow. Firstly, the data generation and bioinformatic workflows are ISO accredited and all bioinformatic tools and processes are version controlled. Secondly, each change in the workflow is validated using a combination of reference material (e.g., Genome-in-a-bottle samples NA24149, NA24143 and NA24385, NA24631) and reanalysis of a representative set of previously analyzed cases with specific genetic aberrations. Recently, we have implemented a continuous quality assurance workflow using the tool MutAcc [39] (Additional file 1: Supplementary Methods). This tool enables simultaneous testing of sensitivity to call several hundred pathogenic variants among our previously diagnosed cases by collecting the underlying reads supporting the pathogenic variants and creating a synthetic genome, on a Genome-in-a-bottle genome backbone, containing all these variants. This synthetic genome can be analyzed upon validation of each change in workflow, as well as at regular intervals, thereby providing the basis for a continuous quality assurance program.

## Results

### Overall statistics

During the period 2015–2019, 3219 patients have been analyzed by WGS within a clinical setting through GMCK-RD. Over time, the number of patients analyzed has increased dramatically (Fig. 1). The distribution for singletons vs trios was 84% compared to 16%, and “phenotype-generated panels” vs “OMIM morbid gene panel” was 92% versus 8%, which altogether illustrates that the vast majority of samples have been analyzed as singleton cases with a phenotype-specific gene panel. Including index cases as well as healthy and affected relatives in total, 4437 WGS samples had been processed through our pipeline by the end of December 2019.

Samples were sequenced to a median of 452 million read pairs (PE 150 bp, SD 192 M read pairs), corresponding to approximately 40x deduplicated mean coverage.

The turnaround time (TAT) for data generation and bioinformatic analysis, measured as the time from extracted nucleic acid until results ready for final clinical interpretation, was median 13 days (min 2, max 43, SD = 5.4 days) (Fig. 1; Additional file 1: Figure S2). Samples sequenced on the HiSeq X platform were processed slightly quicker due to more frequent sequencing starts and sequencing of one sample per lane. TAT above 25 days were often linked to the need to request additional genomic DNA for library preparation. In addition to this, time was also needed for variant interpretation and reporting of the results. In general, there were three priority groups differing in total TATs (from arrival of sample to distribution of a written report) based upon the urgency of the analysis. Regular analyses had a TAT of 1–3 months, priority analyses had a TAT of 2–4 weeks, and acute analyses had a TAT of 4–14 days.

For all panels, the most frequently requested ones were the IEM and neuromuscular and ataxia panels. The number of cases for each panel is shown in Table 1. EP and OMIM morbid gene panels are generally performed as trio analysis as de novo mutations are common causes of disease. Overall, 3750 panel analyses have been performed in the 3219 rare disease cases amounting to 1.09 panels per individual (range 1–3). In 173 cases, data was shared between clinics within GMCK-RD.

The total number of cases that received a molecular diagnosis was 1285, rendering an overall yield of 40% in the study population. The diagnostic rate for singletons versus trios was 34% compared to 36%. Considering the “disease-specific panels” versus “OMIM morbid gene panel,” the diagnostic yield was 35% and 41% respectively. An increase in the diagnostic yield was achieved by reanalysis of the WGS data through updated versions of both MIP and gene panels. Specifically, 16% (130 cases) of the patients analyzed with the gene panels IEM and EP underwent reanalysis resulting in 19% (25 cases) of these receiving a molecular diagnosis. Diagnostic yield varied between 19 and 54% for different clinical entities/panels (Table 1). A total of 8 cases received a dual diagnosis (Additional file 1: Table S5).

### Heterogeneity

Within the group of 1293 positive findings, there was heterogeneity regarding causative genes, and even more so when looking at specific variants (Additional file 3: Table S7). However, some of the genes were recurrent, and also, some of the causative variants proved to be recurring in multiple cases. In total, variants in 754 different disease genes were reported, with the most prevalent ones being *COL2A1* and *FKRP* (*n* = 12 cases per gene) followed by *MECP2* with eleven cases and *DYNC1H1* with ten cases. *COL1A2*, *COL5A1*, *FBN1*, *KCNQ2*, and *STXBP1* (*n* = 9 cases per gene) as well as *ARID1B*, *RYR1*, and *SCN1A* (*n* = 8 cases per gene) were also common findings in our cohort (Additional file 3: Table S7). For the majority of all disease genes (496/754; 66%), reported variants were detected only in one single patient from the study cohort (Additional file 1: Figure S3; Additional file 3: Table S7).

### Recurrent variants

A number of the causative variants were recurrent and thus detected in multiple unrelated individuals. Some of these variants are known founder mutations, such as c.826C>A, p.(Leu276Ile) in FKRP and AAGGG repeat expansion in RFC, which were seen in a homozygous state in twelve individuals with limb-girdle muscle dystrophy (LGMD2I) and in five individuals with CANVAS, respectively, [40] as well as c.1150G>A, p.(Glu384Lys) in TIA1 and c.148G>A, p.(Val50Met) in TTR, which were each detected twice in individuals with the autosomal dominant disorders Welander distal myopathy and hereditary amyloidosis, respectively [41, 42].

In addition to known founder mutations, recurrent variants were detected in seemingly unrelated cases with a non-Swedish origin from the same geographical region. This is exemplified by two individuals with a homozygous nonsense mutation (c.1969G>T, p.(Glu657*)) in *CAPN1*, compatible with a diagnosis of autosomal recessive spastic paraplegia (SPG76).

A few individuals harbored variants that are known to recurrently arise de novo and also segregate in families with autosomal dominant diseases as exemplified by six cases with the common c.694dup, p.(Arg217Profs*8) pathogenic variant in the *PRRT2* gene causing seizures and two individuals with multiple exostoses, carrying variants affecting the coding nucleotide 1018 in *EXT1* (c.1018C>T, p.(Arg340Cys) and c.1018C>G, p.(Arg340Gly) respectively). The c.1018C>G variant was mosaic and only present in 4/33 reads (confirmed by Sanger sequencing). We also found unrelated cases carrying the same rare variant in autosomal dominant disorders, exemplified with *CHD7* (c.2504_2508del, p.(Tyr835Serfs*14)), *IFITM5* (c.-14C>T), *MPZ* (c.418T>A, p.(Ser140Thr)) and *MSH2* (c.942+3A>T). All recurrent variants are listed in Table 2.

**Table 2:** Recurrent variants in the cohort

Gene	Variant	Inheritance	Type of recurrence	Number of cases
*CAPN1*	c.1969G>T, p.(Glu657*)	AR (homozygous)	Novel	2
*DOK7*	c.1124_1127dup, p.(Ala378Serfs*30)	AR	Founder mutation	2 (both cases compound heterozygous)
*ECEL1*	c.494T>C, p.(Leu165Pro)	AR	Founder mutation	2 (one homozygous and one compound heterozygous case)
*EXT1*	c.1018C>G, p.(Arg340Gly) and c.1018C>T, p.(Arg340Cys)	AD	Hotspot mutation	2 (one each)
*FGFR3*	c.1620C>A, p.(Asn540Lys)	AD	Hotspot mutation	2
*FKRP*	c.826C>A p.(Leu276Ile)	AR (homozygous)	Founder mutation	12
*LAMA2*	c.?_4312_4436_?dup	AR (homozygous)	Novel	2
*PRRT2*	c.694dup p.(Arg217Profs*8)	AD	Hotspot mutation	6
*RFC1*	Expansion	AR (homozygous)	Founder mutation	5
*TIA1*	c.1150G>A, p.(Glu384Lys)	AD	Founder mutation	2
*TTR*	c.148G>A, p.(Val50Met)	AD	Founder mutation	2

### CNVs, CGRs, UPD, and STR expansions

Structural variant calling in gene panels was introduced in 2017; genome-wide UPD and STR analyses were added to the WGS pipeline during 2019 (Fig. 2). The introduction was gradual, initially using local variant databases of limited scope and only one variant caller, and early findings were mostly as compound variants with plausible SNVs. Screening was gradually spread to relevant panels, including STRs or genome-wide CNV analysis for relevant indications, with most panels currently adopting at least some triage of non-SNVs. The exact number of cases analyzed for non-SNVs in our cohort is therefore not possible to calculate; however, out of 285 cases explicitly referred for non-SNV screening, 35 (12%) showed non-SNV variants. In total, 64 cases have been reported with a clinical non-SNV or INDEL result. In 45 of those cases (70%), the disease-causing variants were copy number variants (CNVs > 50 bp; 36 deletions and 9 duplications). The findings also include five balanced rearrangements and two complex genomic rearrangements (CGRs). Finally, one case with a maternal UPD of chromosome 7 and ten cases with pathological STR expansions were found. The numbers for each gene panel are still too small to allow interpretation of results of non-SNV/INDEL screening. However, for the initial 100 cases in the NMD and ID panels, respectively, six and 14 pathogenic non-SNVs were detected.

### Mode of inheritance

Of the 1285 positive findings, inheritance could be determined for 870 (68%) (Additional file 3: Table S7). The most common inheritance pattern was autosomal recessive, which was seen in 468 variants (54%) followed by de novo (autosomal dominant as well as X-linked) in 235 variants (27%), inherited autosomal dominant in 107 variants (12%), inherited X-linked in 48 variants (5%), and mitochondrial inheritance in 11 variants (1%) (Additional file 3: Table S7). For the mtDNA variants, inheritance was confirmed to be maternal in 6 cases, 2 of the variants were de novo, and the remaining 3 could not be determined due to lack of maternal samples.

For the remaining findings, inheritance patterns were assumed, due to the fact that parental samples were not—to date—analyzed regarding the genetic finding (*n* = 415). Thus, the total distribution of autosomal dominant disorders (including de novo variants) was 52% (*n* = 669), autosomal recessive disorders 39% (*n* = 504), and X-linked disorders 8% (including de novo variants) (*n* = 101) (Additional file 3: Table S7).

### Confirmation of WGS variants by a secondary method

In clinical practice, Sanger sequencing is typically used for secondary verification of SNVs and INDELs detected by massively parallel sequencing. Although a reliable method, Sanger sequencing is nevertheless an expensive and time-consuming step. To explore if criteria could be established that would allow skipping the Sanger sequencing, a total of 721 variants, where findings had been analyzed with Sanger sequencing, were evaluated retrospectively. Of these, 721 variants, 32 had false-positive results; 31 of the 32 variants were in turn INDELs (incorrect calls mainly due to repetitive sequences or wrong nomenclature regarding the variant). One of the 32 variants was a single base pair substitution with a genotype quality score below GATK’s maximum value of 99. By excluding all INDEL variants and variants with a quality score less than maximum, 503 variants of the total 721 variants remained. Of these, 493 were called with a “pass” using GATK. In addition, it was decided by the GMCK-RD working group that the SNV variant should have at least 20 reads, appear valid upon manual visual assessment using IGV, and not be present in segmental duplication regions (http://genome.ucsc.edu/cgi-bin/hgTrackUi?g=genomicSuperDups). Using these criteria, we aimed to exclude reporting false-positive results, and thereby 484 variants (67%) would not need verification using a secondary method (for summary, see Table 3). A later reanalysis including more variants (data not shown) showed that 64% of our results fulfilled the above criteria and thus did not need secondary verification.

**Table 3:** Criteria for excluding secondary verification of SNV WGS data

- Single base pair substitution with a sequence depth of 20x at that specific position	
- Genotype quality score of 99 (GATK, maximum)	
- Detected with “pass” using GATK	
- Good quality using visual inspection in IGV, or similar software	
- Not present in segmental duplication regions	

### Research analysis and external data sharing

After clinical analysis, there were still cases where there was a high suspicion of an underlying genetic cause that remained unsolved. In these cases, it was possible to perform additional analysis runs through the research pipeline looking at all genes in the genome. By this approach, 17 novel disease genes, inheritance patterns, or mechanisms for disease/pathogenesis were found that have been reported so far [43–59]. To facilitate discovery of new disease genes, GMCK-RD has recently joined international data sharing initiatives, including UDNI, Beacon, and MatchMaker Exchange.

## Discussion

Many large-scale genome sequencing projects are ongoing globally, but clinical implementation is for the most part lagging behind. We describe an integrated approach where the rapid technological development in genomics is harnessed for the benefit of patients with rare genetic diseases, by embedding genomic infrastructure and expertise into healthcare making it available across a broad range of clinical scenarios.

The availability of whole exome and whole genome sequencing has drastically impacted genetic diagnostics, and the clinical genetics specialty is undergoing rapid development. Genetic diagnostics was until recently limited to investigations of chromosome aberrations, by karyotyping or array analysis, and gene by gene sequencing. Consequently, a strong focus has been on conditions like, e.g., unclear malformation syndromes and intellectual disability, together with selected monogenic disease groups where a limited number of underlying disease genes have been defined. Genomics fundamentally changes this scenario. Around 4200 different monogenic disease genes are currently known [2], causing conditions that present across all clinical disciplines, at all ages, and ranging from insidious, chronic, to dramatically acute diseases. The possibility to incorporate WGS in the diagnostic workup across these vastly different clinical situations provides tremendous opportunities, but also poses challenges.

Due to the decentralized structure of Swedish healthcare and the separate governance systems between healthcare and academia, national coordinated initiatives in genomic medicine are complicated. We have not performed a large-scale prospective research study where we have collected patient data that is free for us to investigate in depth. Instead, we describe a bottom-up approach, by which we have truly integrated genome sequencing into real-time clinical investigations, by gradually bringing together different areas of expertise and adding novel components over time. This also underlies our restricted, panel-based approach, where more extensive data mining is not automatically performed in all cases. Instead, we focus on finding genetic variants explaining each patient’s specific clinical situation and avoiding unclear, unanticipated, and irrelevant findings.

The GMCK-RD format has to date enabled more than 3200 rare disease patients access to genomic investigations in a clinical setting providing a diagnosis to more than 1200 individuals. One major challenge with genome sequencing is the high number of variants present per individual with millions of genetic variants generated in each sequenced patient [60]. Managing and interpreting this data in relation to each individual disease presentation requires a highly complex, multidisciplinary workflow. By restricting analyses to rare variants in genes relevant for each patient’s individual disease presentation or inheritance pattern in a family, a manageable number of variants can be generated for evaluation by a diagnostic team. Highly specialized clinicians are important in making the initial patient selection and detailed phenotyping, to help direct the first-line analysis to the most appropriate gene panel and to generate customized, HPO-based panels if necessary. Despite restriction to specific gene panels, variants of unknown clinical significance are common, and no algorithm exists that can precisely predict function and in vivo relevance of most of these. Detailed clinical expertise and complementary diagnostic tests facilitate the assessment of such variants.

The value of rapid, targeted analyses is particularly evident in some disease areas, such as inborn errors of metabolism where specific treatments sometimes can prevent serious complications or death. As one example, in patients with suspected acute-onset IEM, general support is often provided before a definite diagnosis has been established. This can include glucose infusion to block catabolism reducing potentially toxic intermediates and to prevent cellular energy deficiency. However, in pyruvate dehydrogenase deficiency (PDHD), which can cause acute or intermittent encephalopathy with severe neurological sequelae, glucose infusion is detrimental rather than beneficial. PDHD, which can be caused by at least six different genes, should be treated with carbohydrate restriction followed by a ketogenic diet. Dichloroacetic acid can also be beneficial and some patients are thiamine-responsive [61]. Among the patients we report, nine were diagnosed with PDHD. These were previously unsolved cases and the opportunities to significantly improve their clinical outcome had passed. From now on, time to specific treatment can be reduced and the extent of brain damage can be diminished in these patients. Although the full impact measured by improved clinical outcomes will await future clinical follow-up studies, there were examples of direct impact on treatment decisions. These included initiation of a ketogenic diet to patients with pyruvate dehydrogenase deficiency (mutations in *PDHA1*, *PDHB*, *DLD*), AGC1 deficiency (mutations in *SLC25A12*), and GLUT1 deficiency (mutations in *SLC2A1*). Thiamine and biotin treatment in biotin- or thiamine-responsive encephalopathy (mutations in *SLC19A3*), folinic acid in cerebral folate deficiency (mutations in *FOLR1*), and creatine in cerebral creatine deficiency (mutations in *SLC6A8*) are other examples. The genetic diagnoses influenced the choice of antiepileptic drugs in many cases. Valproate treatment has been avoided in patients with *POLG* mutations, who may experience serious side effects of this drug. Sodium channel blockers have been avoided in *SCN1A*-related cases (loss-of-function variants) but preferred in early-onset *SCN2A* and *SCN8A* epilepsy (gain-of-function variants). Transdermal nicotine treatment was successfully used in a *CHRNA4*-related case and carbamazepine in *KCNQ2* and *PRRT2* epilepsy.

Human genetic variation is extremely diverse, ranging from small variants affecting single base pairs to large structural variants affecting thousands or millions of nucleotides. Novel types of pathogenic variants affecting coding and non-coding regions are expected to be continuously discovered, and combined effects of different variants will successively be understood. An environment that includes continuous development ensures incorporation of novel features into the workflow as our understanding of disease genetics expands and novel methodologies become available, enabling continuous improvement of the diagnostic yield in this rapidly developing field. This is the way we have established and gradually improved our workflow and bioinformatics pipeline. The possibility of analyzing not only SNVs and INDELS but also CNVs, balanced structural variants, short tandem repeats, and stretches of homozygosity (e.g., from UPD) is a major advantage of WGS compared to exome analysis. We have recently shown that WGS has a high detection rate of both balanced and unbalanced structural variants [21]. In the data shown here, only a fraction of cases have been assessed for structural variants and UPD. However, the increased diagnostic yield by adding those callers (7.5%) is remarkable and shows promise of even higher utility of WGS in the future.

By working stepwise, using targeted panels as a first-line test, consultations and data sharing between complementary teams focusing on different disease groups in the next step, and opening up the whole genome in cases that still remain without a diagnosis, the combined value of rapid, restricted, highly specialized investigations and broad, genetic screening can be achieved. Gene discovery is also enabled, resulting in elucidation of novel pathogenetic mechanisms. In GMCK-RD, a number of novel genes have been identified, resulting in improved biological understanding of disease mechanisms and better patient care as exemplified by *KAT6A* (intellectual disability) [43], *SLC12A5* (epilepsy of infancy) [55], and *MIR140* (skeletal dysplasia) [52].

In addition to disease gene discovery, there is also an intense development of novel treatments in the rare disease area, in the form of, e.g., recombinant enzymes and other biologicals, small molecule drugs, antisense technologies, gene therapy, and genome editing/cell therapies. The use of such novel treatments will be critically dependent on accurate diagnostics, both in order to identify patients who are likely to benefit and to avoid use by those who will not.

Here we show that by applying a standardized workflow for clinical WGS in an integrated clinical-academic setting we achieve solve rates of 19–54% across a broad area of phenotypic sub-groups. The current challenges for large-scale use of WGS in healthcare involve both practical and legal issues that need to be clarified and limitations of crucial resources such as OMIM [2] and HPO [36]. The need for updated gene-phenotype databases cannot be over-emphasized. Finally, with increasing demand (128% increase of samples between 2016 and 2019; Fig. 1), it is important to build sustainable structures bridging healthcare and academia that are not critically dependent on critical individuals struggling to collaborate across silos. Rather, it is essential to establish novel organizational structures that support the integrated concept, bringing cutting-edge technology all the way to treating clinicians, who are critical for patient selection, rapid interpretation of results, and translation into individualized clinical management.

The experience and findings from the implementation in the regional Stockholm healthcare described here are now being used to facilitate a national implementation of WGS-based rare disease diagnostics through the Swedish national genome initiative Genomics Medicine Sweden. Despite regional differences in technical infrastructure and clinical expertise, we expect the Swedish healthcare system to implement WGS systematically across the different healthcare regions and disease categories.

## Conclusions

We show that by building an environment where highly specialized physicians work closely together with trained clinical molecular geneticists and experts in laboratory medicine, genomics, and bioinformatics, an environment of continuous learning has been created. This generates strong synergies and puts clinical medicine in a much better position to keep pace with the ongoing rapid scientific and technological developments. As this requires fundamentally novel ways of working across disciplines both within healthcare and between healthcare and academia, efforts are needed to reorganize academic medicine to work less in silos and enable sharing of data and expertise. If this can be achieved and the concept can be consolidated and spread, we are taking decisive steps towards precision medicine.

Clinical WGS has turned out to be a true game changer in the rare disease area. During the first years of GMCK-RD’s activities, > 1200 patients received specific molecular diagnoses that could not have been achieved in the same timeframe before MPS technology was developed. This has had an impact on affected patients and their families, by providing explanations for their diseases and ending diagnostic odysseys. In addition, patients and their families have been offered genetic counseling, prognostic information, and specific treatments.

## Supplementary Information

**Additional file 1: Supplementary methods**, **Table S1.** List of SNPs used for genotyping, **Table S2.** In house developed open source software used, **Table S3.** Publicly available open source software used, **Table S4.** Publicly available databases used, **Table S5.** Cases with a dual diagnosis. **Figure S1.** Steps performed in the MIP rare disease pipeline, **Figure S2.** Turnaround time per quarter, **Figure S3.** Statistics regarding number genes and patients.

**Additional file 2: Table S6.** Panel gene lists.

**Additional file 3: Table S7.** List of genes and variants reported in cases.

## Data Availability

Detected pathogenic variants have been submitted to ClinVar, submission number SUB8639822, https://www.ncbi.nlm.nih.gov/clinvar/?term=SUB8639822 [62]. The ethical approval did not permit sharing of WGS data, and the in-house databases used in this article are not publicly available. Developed software (*Cgbeacon*, https://github.com/Clinical-Genomics/cgbeacon [63]; Genmod, https://github.com/moonso/genmod [31]; *MutAcc*, https://github.com/Clinical-Genomics/mutacc [39]; *Patientmatcher*, https://github.com/Clinical-Genomics/patientMatcher [64]; *Rhocall*, https://github.com/dnil/rhocall [65]; Scout, https://github.com/Clinical-Genomics/scout [35]; *Stranger*, https://github.com/moonso/stranger [23]), public databases, and open-source software used are listed in Additional file 1: Table S2.

## Abbreviations

WGS: Whole genome sequencing
SNV: Single nucleotide variant
CNV: Copy number variant
SV: Structural variant
HPO: Human Phenotype Ontology
STR: Short tandem repeat
INDELs: Small insertions and deletions
MPS: Massively parallel sequencing
