# Supplementary material for: Integration of whole genome sequencing into a healthcare setting: high diagnostic rates across multiple clinical entities in 3219 rare disease patients
Source: Genome Med. 2021 Mar 17;13:40. doi: 10.1186/s13073-021-00855-5 (PMC7968334; doi:10.1186/s13073-021-00855-5)
Supplement: Supplementary file 1 — Additional file 1: Supplementary methods, Table S1. List of SNPs used for genotyping, Table S2. In house developed open source software used, Table S3. Publicly available open source software used, Table S4. Publicly available databases used, Table S5. Cases with a dual diagnosis. Figure S1. Steps performed in the MIP rare disease pipeline, Figure S2. Turnaround time per quarter, Figure S3. Statistics regarding number genes and patients. [file 13073_2021_855_MOESM1_ESM.docx]

**Additional file 1**

**Supplementary methods**

**Data sharing**

**ClinVar**

Sharing disease causative variants with the global research and diagnostic community, allows the shortening of patients’ diagnostic odyssey. Scout holds all necessary genotype, phenotype, and meta data to autofill a ClinVar-submission of validated causative variant(s) for manual review by the investigator. Further details for Scout can be found below. After curating the assembled ClinVar information the investigator can download a file ready for ClinVar submission via the ClinVar submission portal. Hence, the ClinVar submission process is made significantly more robust, easier and faster.

**Beacon Network**

When analyzing the genome of rare disease patients, it is vital to determine the allele frequency of a variant under investigation in any population or sample cohort. Therefore, GMCK-RD is part of Beacon network, which is a global search engine for genetic variants via the Clinical Genomics ScilifeLab Beacon (See Bioinformatic software - Cgbeacon). Thus, request from the global network to our internal variant collection is handled by Cgbeacon (<https://github.com/Clinical-Genomics/cgbeacon>), while outgoing request for each variant in the interpretation to the Beacon network is performed from the Scout interface.

**Matchmaker Exchange**

To prove a variant to be the disease-causing variant is often difficult because it is hard to find other patients with identical mutations or mutations within the same gene or pathway with similar phenotypes. To facilitate, finding the “other” patients with the same rare disorder, we integrated GMCK-RD as a node in the Matchmaker Exchange (MME) network via Scout and the Clinical Genomics Patientmatcher server (<https://github.com/Clinical-Genomics/patientMatcher>). Scout handles MME requests by communicating with the Patientmatcher server, which in turn is connected to MME network. Scout holds both genotype and phenotype information on each patient, which enables investigators to easily submit candidate genes/variants and/or phenotypes the MME network via the Scout interface.

**Bioinformatic Software**

**Chanjo**

Chanjo enables efficient sequence data coverage analysisacross sets of genomic intervals and biological features. Chanjo operates on coverage reports generated from tools such as sambamba and bedtools. The coverage analysis can be conveniently broken down to different levels of abstraction e.g., genes, transcripts. The aggregated data is stored in a database for interrogation, long-term storage and downstream report generation. Chanjo is an open source software that is distributed under the MIT license, and is available at <https://github.com/Clinical-Genomics/chanjo>.

**Cgbeacon**

Cgbeacon an Elixir-based beacon connected to a MySQL database for storing variants. Cgbeacon will parse variants from a VCF file using either/or a list of gene panels or quality thresholds for inclusion in the beacon server. Cgbeacon is licensed under the MIT license, and is available at <https://github.com/Clinical-Genomics/cgbeacon>.

**Genmod**

Genmod is a comprehensive tool for annotating patterns of inheritance in arbitrary sized pedigrees. Genmod also uses a weighted sum model to prioritize any type of variant that adhere to the VCF format based on a range of annotations specified by the user. Genmod operates on standard bioinformatic formats like VCF and PED and can annotate genetic models in arbitrary pedigrees for both phased and non-phased data. Genmod is distributed under a BSD-like license and is available here <https://github.com/moonso/genmod>.

**LoqusDB**

LoqusDB is a tool to solve the challenge of keeping track of any type of variant observations from genome sequencing data, including SNVs, INDELs and SVs. LoqusDB was designed to handle a large flow of WGS samples and unlike other solutions, samples can be added continuously to the database without rebuilding it. LoqusDB is an open source software that is distributed under the MIT license, and available here <https://github.com/moonso/loqusdb>.

**MIP**

MIP performs whole genome or target region analysis of sequenced single-end and/or paired-end reads from the Illumina platform in fastq(.gz) format to generate annotated ranked potential disease-causing variants. MIP performs QC, alignment, coverage analysis, variant discovery and annotation, sample checks as well as ranking the found variants according to disease potential with a minimum of manual intervention. MIP is compatible with Scout for visualization of identified variants.

- MIP rare disease DNA analyses single nucleotide variants (SNVs), insertions and deletions (INDELs) and structural variants (SVs), Fig. S1.
- MIP rare disease RNA analyses mono allelic expression, fusion transcripts, transcript expression and alternative splicing.
- MIP rare disease DNA vcf rerun performs re-runs starting from BCFs or VCFs.

MIP is licensed under the MIT license, and is available at <https://github.com/Clinical-Genomics/MIP>

Fastq data: Sequence reads in fastq format

Sequence QC:

1. QC of sequence reads using Fastqc

Alignment:

1. Align sequence reads to the human genome reference using BWA mem
2. Aligned reads are coordinate sorted using sambamba sort

Alignment Recalibration:

1. Merge all sorted BAM files using Picardtools
2. Mark duplicate in BAM file using Picardtools
3. Recalibrate base quality score using GATK

Alignment QC:

1. Collect multiple classes of alignment metrics using Picardtools
2. Collect hybris-selection metrics using Picardtools
3. Analyse gender using coverage information on X and Y chromosomes using Chanjo sex-check
4. Collect coverage across gene panels for input into a Chanjo database using sambamba depth
5. Downsample Mitochondrial reads to enable viewing in IGV.js via Scout.
6. Calculate coverage of chromosomes in bins using TIDDIT

SV Variant Calling:

1. Call SV using TIDDIT
2. Call SV using Manta
3. Call copy number variants using CNVnator

SNV/INDEL Variant Calling:

1. Call SNVs and INDELs via local re-assembly of haplotypes using GATK
2. Perform joint genotyping on one or more samples pre-called with HaplotypeCaller using GATK
3. Gathers multiple VCF files from a scatter operation into a single VCF file using GATK
4. GATK tool using machine learning to model the technical profile of variants in a training set and uses that to filter out probable artifacts from the callset

STR Variant Calling:

1. Call and estimate repeat sizes on candidate list of genetic regions using ExpansionHunter

SMN Variant Calling:

1. Call the copy number of full-length SMN1, full-length SMN2, as well as SMN2Δ7–8 (SMN2 with a deletion of Exon7-8) using SMNCopyNumberCaller

SV Annotation:

1. Merge VCFs from different callers using GATK
2. Annotate variants effects of variants using the Variant Effect Predictor tool
3. Split vcf records on transcript level into an in-silico clinical screen file and research file
4. Annotate inheritance patterns and generate a prioritization score for each variant

SNV/INDEL Variant Annotation:

1. Call regions of homozygosity and make tentative UPD calls
2. Normalise and decompose vcf records per alternative allele
3. Annotate VCF with allele frequencies from public and local databases
4. Calculate CADD score for indels
5. Call UPD regions and create images of the results
6. Annotate VCF using the Variant Effect Predictor tool
7. Split vcf records on transcript level into an in-silico clinical screen file and research file
8. Annotate inheritance patterns and generate a prioritization score for each variant

STR Variant Annotation:

1. Annotate variants with the pathologic implications of the repeat sizes

Variant QC:

1. Compare familial-relationships, ethnicity and sexes as reported by user, with those inferred from the VCF
2. Compare familial-relationships and sexes as reported by user, with those inferred from the VCF
3. Check for mendelian errors and correct father in trios
4. Variant comparison at the haplotype level
5. General-purpose tool for variant evaluation

Analysis QC:

1. Set state of MIP analysis and provide information for evaluation of analysis qc metrics and analysis run status
2. Aggregate results from bioinformatics analyses across many samples into a single report
3. Collect all binary version used in an analysis
4. Compare and report Mutacc generated continuous evaluation genomic background sets
5. Pass or fail an analysis run depending on several criterias
6. Status file of analysis run progress and exist codes

**MutAcc**

MutAcc is a tool to artiﬁcially enrich well-known MPS datasets with clinical variants obtained from empirical data. MutAcc has an interface that continuously imports data to the database from the decision support tool, Scout and provides the functionality to export the created "synthetic" data into a genomic background data set that can be used in continuous validation of bioinformatic tools and workflows using real clinical data*.*

MutAcc is licensed under the MIT license, and is available here <https://github.com/Clinical-Genomics/mutacc>.

**patientMatcher**

patientMatcher is a matchmaker exchange server using mongoDB. The phenotype scoring algorithm of patientMatcher is based on genotype and phenotype (HPO-terms). patientMatcher is licensed under the MIT License, and is available here <https://github.com/Clinical-Genomics/patientMatcher>.

**Scout**

*Scout* is an open-source decision support framework for clinical diagnostics with graphical user interface (GUI) for MPS associated data. Scout lets users organize, collaborate, share, inspect, filter, annotate, explore and analyze variant calls via a standard web browser. It provides a unified and intuitive interface for all loaded resources and can be used by anyone without computer programming literacy. Scout is distributed under the BSD 3-Clause "New" or "Revised" License, and is available here <https://github.com/Clinical-Genomics/scout>.

**Stranger**

Stranger annotates STR variants with pathologic implications of different repeat sizes. The repeat size intervals that come with the package are manually curated from the literature since there is yet no centralized source for this type of information. Stranger works on theoutput from Expansionhunter which will annotate each variant with the number of times that a repeat has been seen in the alignment file of each individual and add a repeat id. Stranger is distributed under the MIT license and is available here <https://github.com/moonso/stranger>.

**TIDDIT**

TIDDIT (Eisfeldt et al TIDDIT) is a locally developed structural variant caller, using discordant read pairs, split reads and to a lesser degree read depth to call balanced and unbalanced structural variation. It is distributed under the GNU general public license version 3.0 and is available here <https://github.com/J35P312/TIDDIT>.

**SVDB**

SVDB (Eisfeldt et al TIDDIT) is a locally developed structural variant merging, database, and frequency annotation tool. Variant clustering is similar to TIDDIT. It is distributed under the GNU general public license version 3.0 and is available here <https://github.com/J35P312/SVDB>.

**VCF2cytosure**

VCF2cytosure converts a VCF with structural variant calls into the .CGH format used by CytoSure Interpret, OGT (Oxford Gene Technology). Read depth in bins provided in a bed file, e.g. by TIDDIT, is also visualized in the CGH file. This allows a clinical operator familiar with the CytoSure tool to evaluate structural variation from wgs data. Vcf2cytosure is distributed under the MIT license, and is available here <https://github.com/NBISweden/vcf2cytosure>.

**Rhocall**

Rhocall calls stretches of homozygosity and annotates variants in a VCF with this information. It is distributed under the GNU GPL 3.0 license and is available here <https://github.com/dnil/rhocall>.

**Supplementary tables**

**Table S1:** The SNP list used for ID typing using genome build grch37

| #CHROM | POS | ID | REF | ALT |
| --- | --- | --- | --- | --- |
| 1 | 44072018 | rs1065772 | C | T |
| 1 | 183072590 | rs2296288 | T | C |
| 1 | 212619339 | rs4804 | T | C |
| 2 | 85553784 | rs1044973 | C | T |
| 2 | 235950187 | rs1469375 | T | C |
| 3 | 48474249 | rs2279077 | G | A |
| 3 | 48508585 | rs11797 | T | C |
| 4 | 73956736 | rs6855349 | G | A |
| 5 | 96498783 | rs8654 | G | A |
| 5 | 112176325 | rs42427 | G | A |
| 6 | 11213832 | rs3734404 | T | C |
| 6 | 26545632 | rs4871 | G | A |
| 6 | 56471995 | rs16888055 | C | T |
| 6 | 138413269 | rs648396 | A | G |
| 6 | 157405930 | rs3734441 | G | A |
| 7 | 1542697 | rs3752714 | G | A |
| 7 | 77423574 | rs7953 | G | A |
| 7 | 151074296 | rs2159158 | A | G |
| 8 | 22432377 | rs710098 | C | A |
| 8 | 125498547 | rs3812471 | C | T |
| 9 | 712599 | rs11789987 | C | T |
| 9 | 139391636 | rs2229974 | G | A |
| 10 | 24833137 | rs2250411 | C | T |
| 12 | 993930 | rs7300444 | C | T |
| 12 | 48539482 | rs8716 | T | G |
| 12 | 133236000 | rs5744857 | C | T |
| 13 | 28239970 | rs14105 | G | A |
| 14 | 34985645 | rs7797 | G | A |
| 14 | 55817708 | rs10144418 | T | C |
| 15 | 55653031 | rs501231 | A | G |
| 15 | 93567864 | rs12906163 | A | C |
| 15 | 100252805 | rs325400 | G | T |
| 16 | 2285357 | rs26840 | C | T |
| 16 | 15818141 | rs2075511 | A | C |
| 16 | 29998200 | rs4077410 | A | G |
| 16 | 85009970 | rs7195377 | A | G |
| 17 | 2203453 | rs216193 | A | G |
| 17 | 27869968 | rs507577 | G | A |
| 17 | 40125864 | rs2070106 | G | A |
| 17 | 55183203 | rs2230772 | T | C |
| 17 | 71197748 | rs1037256 | G | A |
| 17 | 76799898 | rs9302885 | A | G |
| 19 | 4818378 | rs7255265 | T | C |
| 19 | 11303943 | rs3745681 | A | G |
| 19 | 58929052 | rs3764535 | G | A |
| 20 | 48522585 | rs492702 | G | A |
| 21 | 16340289 | rs2229741 | C | T |
| 22 | 18209613 | rs4488761 | A | G |
| X | 47444985 | rs4898 | T | C |
| X | 102842041 | rs11010 | T | C |

**Table S2**: In house developed open source software used

| *Software* | *Description* | *Reference* | *Date* |
| --- | --- | --- | --- |
| *Chanjo* | *Coverage database* | https://github.com/Clinical-Genomics/chanjo | *2019-12-31* |
| *Cgbeacon* | *Beacon server* | https://github.com/Clinical-Genomics/cgbeacon | *2019-12-31* |
| Genmod | *Annotation, Prioritization* | https://github.com/moonso/genmod | *2019-12-31* |
| *LoqusDB* | *Observation count database* | https://github.com/moonso/loqusdb | *2019-12-31* |
| *MIP* | *Analysis Workflow* | https://github.com/Clinical-Genomics/MIP | *2019-12-31* |
| *MutAcc* | *Validation* | https://github.com/Clinical-Genomics/mutacc | *2019-12-31* |
| *Patientmatcher* | *MME server* | <https://github.com/Clinical-Genomics/patientMatcher> | *2019-12-31* |
| *Rhocall* | *Homozygosity calling and annotation* | <https://github.com/dnil/rhocall> | *2019-12-31* |
| Scout | *Decision support* | <https://github.com/Clinical-Genomics/scout> | *2019-12-31* |
| *Stranger* | *STR annotation* | <https://github.com/moonso/stranger> | *2019-12-31* |
| SVDB | *SV database* | [*https://github.com/J35P312/SVDB*](https://github.com/J35P312/SVDB) | *2019-12-31* |
| TIDDIT | *SV caller* | <https://github.com/J35P312/SVDB> | *2019-12-31* |
| VCF2Cytosure | *VCF converter* | https://github.com/NBISweden/vcf2cytosure | *2019-12-31* |

**Table S3:** Publicly available open source software used

| *Software* | *Description* | *Reference* | *Date* |
| --- | --- | --- | --- |
| *Bedtools* | *Genome analysis toolkit* | *https://github.com/arq5x/bedtools2* | *2019-12-31* |
| *BWA* | *Aligner* | https://github.com/lh3/bwa | *2019-12-31* |
| *Cadd-scripts* | *Annotation* | https://github.com/kircherlab/CADD-scripts | *2019-12-31* |
| *Cnvnator* | *Variant caller* | https://github.com/abyzovlab/CNVnator | *2019-12-31* |
| *Expansionhunter* | *Variant caller* | https://github.com/Illumina/ExpansionHunter | *2019-12-31* |
| *Fastqc* | *Quality control* | https://www.bioinformatics.babraham.ac.uk/projects/fastqc/ | *2019-12-31* |
| *GATK* | *Genome analysis toolkit* | https://github.com/broadinstitute/gatk/ | *2019-12-31* |
| *Htslib* | *Parsing sequencing data formats* | http://www.htslib.org/ | *2019-12-31* |
| *Manta* | *Variant caller* | https://github.com/Illumina/manta | *2019-12-31* |
| *Multiqc* | *Quality control* | https://github.com/ewels/MultiQC | *2019-12-31* |
| *Peddy* | *Quality control* | https://github.com/brentp/peddy | *2019-12-31* |
| *Picard* | *Alignment parsing and quality control* | https://github.com/broadinstitute/picard | *2019-12-31* |
| *Rtg-tools* | *Variant evaluation* | https://github.com/RealTimeGenomics/rtg-tools | *2019-12-31* |
| *Plink* | *Quality control* | https://github.com/chrchang/plink-ng | *2019-12-31* |
| *Sambamba* | *Alignment parsing* | https://github.com/biod/sambamba | *2019-12-31* |
| *Smncopynumbercaller* | *Variant caller* | https://github.com/Illumina/SMNCopyNumberCaller | *2019-12-31* |
| *Vcfanno* | *Annotation* | https://github.com/brentp/vcfanno | *2019-12-31* |
| *VEP* | *Annotation* | https://github.com/Ensembl/ensembl-vep | *2019-12-31* |
| *Vt* | *Vcf parsing* | https://github.com/atks/vt | *2019-12-31* |

**Table S4:** Publicly available databases used

| *Database* | *Description* | *Reference* | *Date* |
| --- | --- | --- | --- |
| *CADD* | *Deleteriousness annotation* | https://krishna.gs.washington.edu/download/CADD/v1.4/GRCh37/whole_genome_SNVs.tsv.gz | *2019-12-31* |
| *Clinvar* | *Human variation and phenotypes annotation* | ftp://ftp.ncbi.nlm.nih.gov/pub/clinvar/vcf_GRCh37/ | *2019-12-31* |
| *Dbnsfp* | *Variant annotation* | ftp://dbnsfp:dbnsfp@dbnsfp.softgenetics.com/dbNSFPv3.5a.zip | *2019-12-31* |
| *GATK* | *Human Reference Decoy 5* | https://storage.googleapis.com/gatk-legacy-bundles/b37/human_g1k_v37_decoy.fasta.gz | *2019-12-31* |
| *GATK* | *Variant calls* | <https://storage.googleapis.com/gatk-legacy-bundles/b37/1000G_phase1.indels.b37.vcf.gz>  <https://storage.googleapis.com/gatk-legacy-bundles/b37/1000G_omni2.5.b37.vcf.gz>  <https://storage.googleapis.com/gatk-legacy-bundles/b37/hapmap_3.3.b37.vcf.gz>  <https://storage.googleapis.com/gatk-legacy-bundles/b37/dbsnp_138.b37.vcf.gz>  https://storage.googleapis.com/gatk-legacy-bundles/b37/Mills_and_1000G_gold_standard.indels.b37.vcf.gz | *2019-12-31* |
| *Giab* | *Gold standard truth set* | <ftp://ftp-trace.ncbi.nlm.nih.gov/giab/ftp/release/NA12878_HG001/NISTv3.3.2/GRCh37/>  <ftp://ftp-trace.ncbi.nlm.nih.gov/giab/ftp/release/AshkenazimTrio/HG004_NA24143_mother/NISTv3.3.2/GRCh37/>  <ftp://ftp-trace.ncbi.nlm.nih.gov/giab/ftp/release/AshkenazimTrio/HG002_NA24385_son/NISTv3.3.2/GRCh37/>  <ftp://ftp-trace.ncbi.nlm.nih.gov/giab/ftp/release/ChineseTrio/HG005_NA24631_son/NISTv3.3.2/GRCh37/> | *2019-12-31* |
| *Gnomad* | *Frequency annotation and constraint* | <https://storage.googleapis.com/gnomad-public/release/2.1.1/vcf/genomes/gnomad.genomes.r2.1.1.sites.vcf.bgz>  <https://storage.googleapis.com/gnomad-public/papers/2019-sv/gnomad_v2_sv.sites.vcf.gz>  https://storage.googleapis.com/gnomad-public/release/2.1.1/constraint/gnomad.v2.1.1.lof_metrics.by_gene.txt.bgz | *2019-12-31* |
| *Spidex* | *Splice annotation* | Not available | *2019-12-31* |
| *Swegen* | *Frequency annotation* | https://swefreq.nbis.se/dataset/SweGen | *2019-12-31* |
| *UCSC* | *Segmental Duplications* | http://hgdownload.cse.ucsc.edu/goldenPath/hg19/database/genomicSuperDups.txt.gz | *2019-12-31* |
| *VEP Cache* | *Variant annotation* | ftp://ftp.ensembl.org/pub/release-97/ | *2019-12-31* |
| *VEP Plugins* | *Variant annotation* | https://github.com/Ensembl/VEP_plugins | *2019-12-31* |

**Table S5:** Cases with a dual diagnosis

| **Case** | **Genes** |
| --- | --- |
| 1 | AP3B2, TBCD |
| 2 | ACADS, COL3A |
| 3 | FLAD1, CPT2 |
| 4 | MMACHC, UGT1A1 |
| 5 | GABRG2, CHD2 |
| 6 | LAMA1, NDUFV2 |
| 7 | TMEM199, PYGL |
| 8 | MOCS2, OPLAH |

**Supplementary figures**


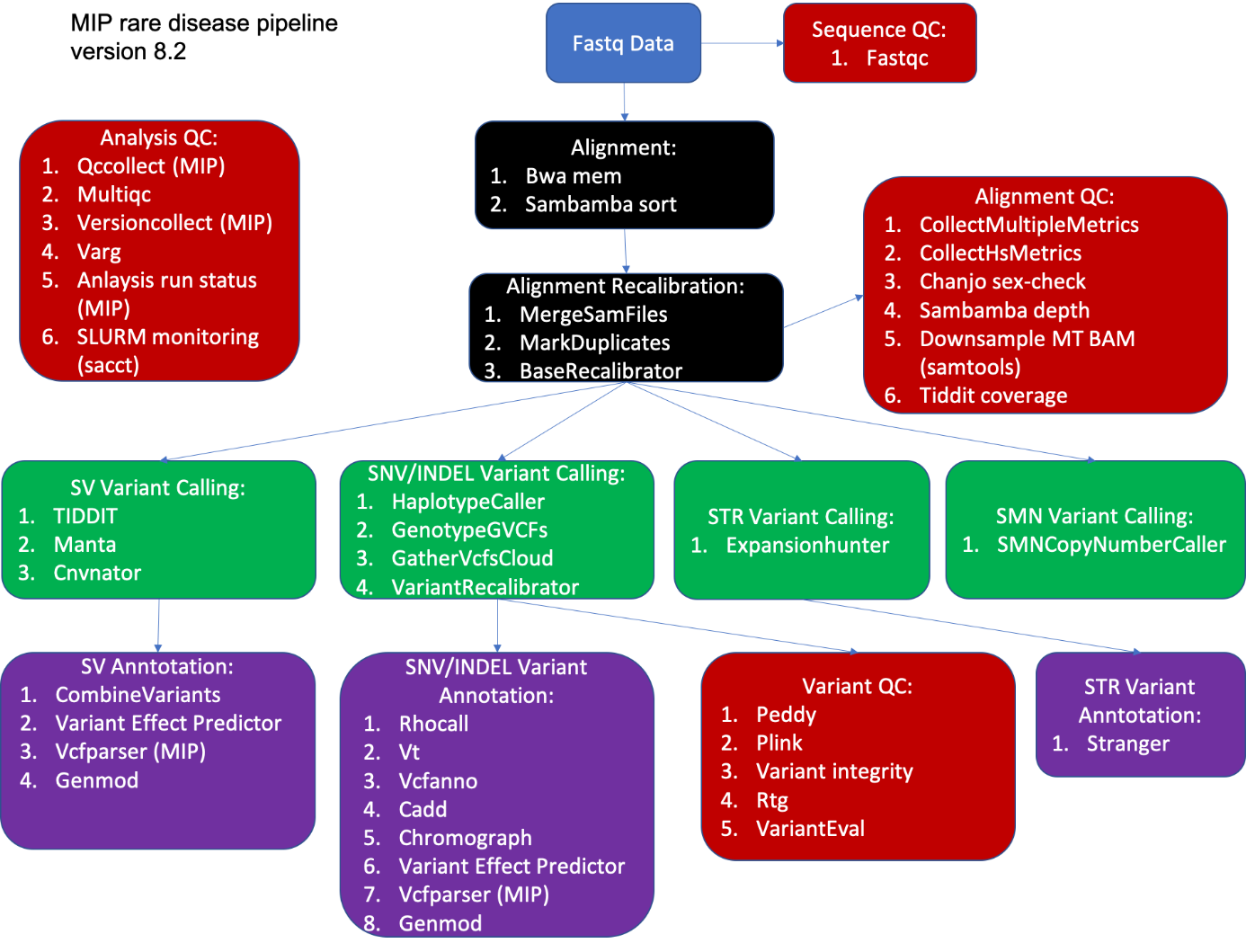


**Fig. S1**. Steps performed in the MIP rare disease pipeline

Quarter

Turnaround time

**Fig. S2**: Turnaround time per quarter

The turnaround time in days for data generation and bioinformatic analysis per quarter.


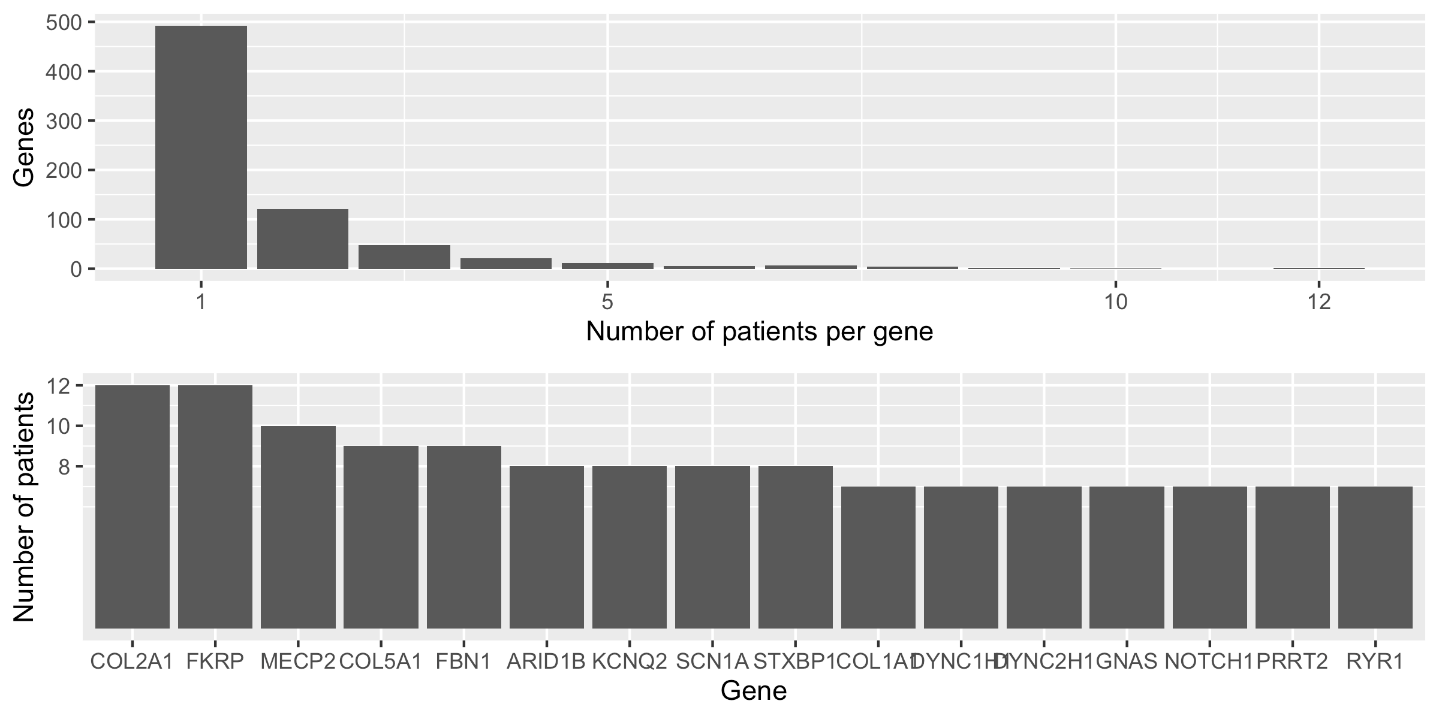


**Fig. S3**: Statistics regarding number genes and patients

A) Number of patients with a disease-causing variant per gene. B) The number patients identified with disease-causing variants in the 16 most frequently mutated genes.
